# Fast and robust shape diameter function

**DOI:** 10.1371/journal.pone.0190666

**Published:** 2018-01-26

**Authors:** Shuangmin Chen, Taijun Liu, Zhenyu Shu, Shiqing Xin, Ying He, Changhe Tu

**Affiliations:** 1 Faculty of Electrical Engineering and Computer Science, Ningbo University, Ningbo, Zhejiang, China; 2 School of Information Science and Engineering, Ningbo Institute of Technology, Zhejiang University, Ningbo, Zhejiang, China; 3 School of Computer Science and Technology, Shandong University, Qingdao, Shandong, China; 4 School of Computer Engineering, Nanyang Technological University, Singapore; Università di Roma, ITALY

## Abstract

The shape diameter function (SDF) is a scalar function defined on a closed manifold surface, measuring the neighborhood diameter of the object at each point. Due to its pose oblivious property, SDF is widely used in shape analysis, segmentation and retrieval. However, computing SDF is computationally expensive since one has to place an inverted cone at each point and then average the penetration distances for a number of rays inside the cone. Furthermore, the shape diameters are highly sensitive to local geometric features as well as the normal vectors, hence diminishing their applications to real-world meshes which often contain rich geometric details and/or various types of defects, such as noise and gaps. In order to increase the robustness of SDF and promote it to a wide range of 3D models, we define SDF by offsetting the input object a little bit. This seemingly minor change brings three significant benefits: First, it allows us to compute SDF in a robust manner since the offset surface is able to give reliable normal vectors. Second, it runs many times faster since at each point we only need to compute the penetration distance along a single direction, rather than tens of directions. Third, our method does not require watertight surfaces as the input—it supports both point clouds and meshes with noise and gaps. Extensive experimental results show that the offset-surface based SDF is robust to noise and insensitive to geometric details, and it also runs about 10 times faster than the existing method. We also exhibit its usefulness using two typical applications including shape retrieval and shape segmentation, and observe a significant improvement over the existing SDF.

## Introduction

The shape diameter function (SDF) is a scalar function defined on a closed manifold surface, which measures the neighborhood diameter of the object at each point and is able to capture the object’s volumetric shape locally. Mathematically, it relates to the medial axis transform [1]. To compute the SDF at a given point *p* on a closed surface *S*, Gal et al. [2] suggested placing an inward cone rooted at *p*, calculating the penetration distances of several rays inside the cone, and then taking the weighted average of these distances as the approximation of the shape diameter at *p*. Whether theoretically or experimentally, SDF is invariant to rigid transformations and oblivious to pose changes. Thanks to these favorable properties, it is widely used in shape analysis, retrieval and segmentation [2–6].

**Fig 1:**
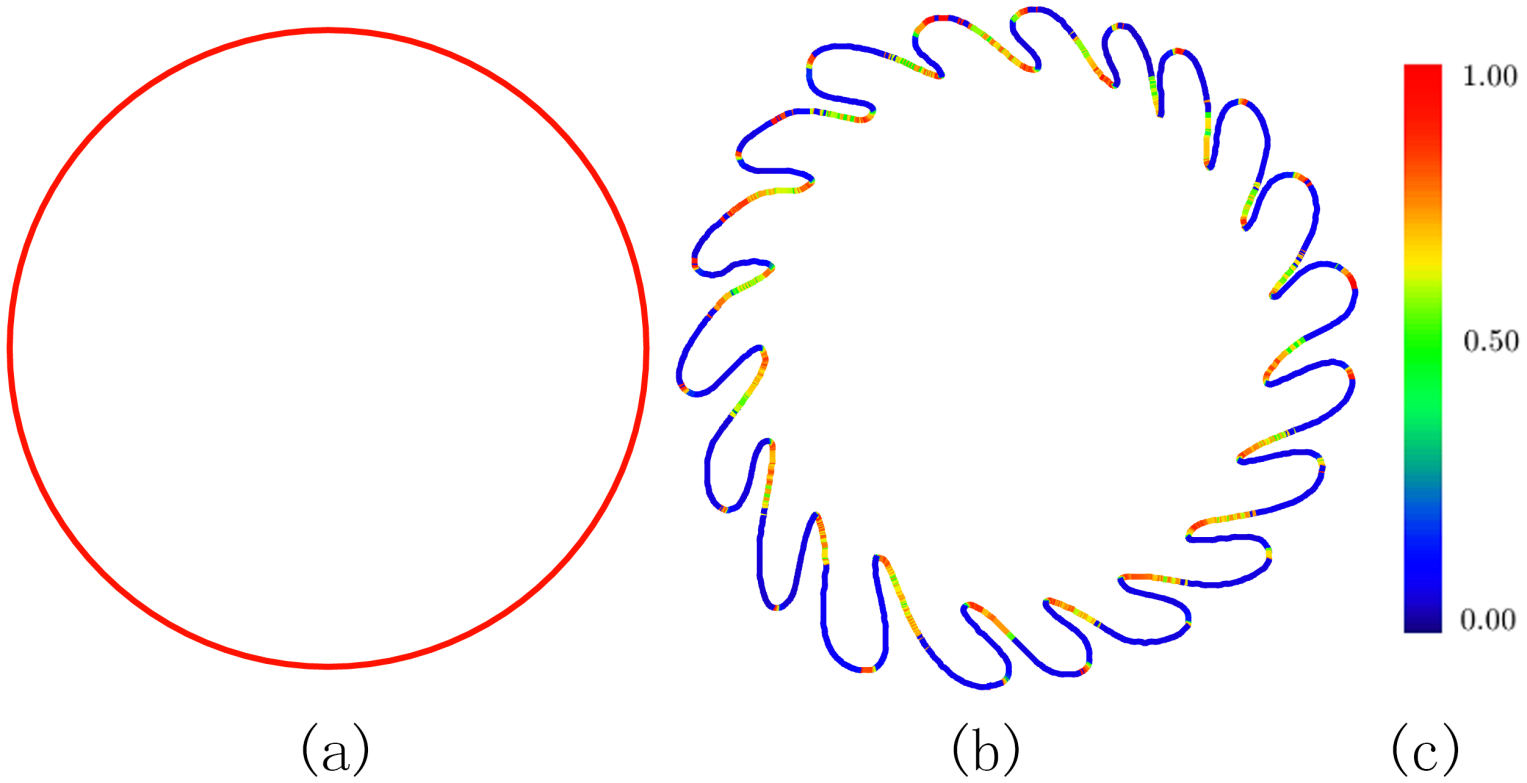
SDF is sensitive to local geometric details. (a) The SDF on a unit circle is a constant function. (b) Adding geometric details to the boundary will produce a completely different SDF.

The SDF in its original form, however, has several disadvantages. First, SDF is highly sensitive to local geometric changes, hereby it does not work for shapes with rich geometric details. As is shown in Fig 1, the SDF on a unit circle is a constant function but has a significant change when we add local geometric details to the boundary. Second, SDF heavily depends on normal vectors and thus one cannot obtain a reliable SDF on a noisy model without preprocessing (e.g., denoising). Third, SDF is not well defined on broken meshes (e.g., point clouds). Last but not the least, computing SDF is computationally expensive since one has to generate tens of rays, instead of a single ray, inside the inward normal cone to estimate the weighted average of penetration distances.

**Fig 2:**
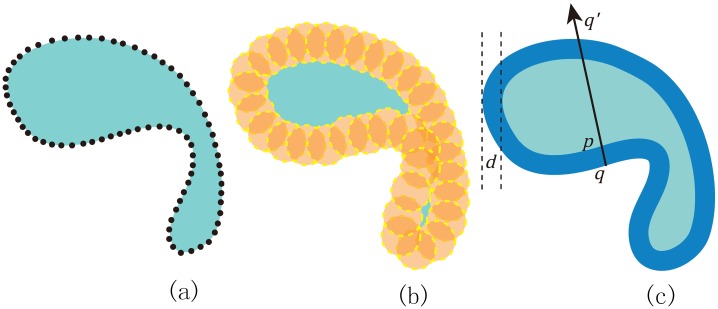
Defining SDF based on the offset surface. (a) The input point cloud. (b) We place a sphere of radius *r* at each point and get the exterior envelope of all spheres. (c) The SDF at a point *p* is determined by ‖*qq*′‖ − 2*r*, where *q* and *q*′ are the two intersection points between the straight line along *p*’s normal vector and the offset surface.

This paper aims at improving the robustness of SDF and promoting it to a wide range of 3D models. Towards this goal, we define SDF based on the offset surface of the input object. Let us denote by *S* the input mesh or point clouds. For each vertex or point, we place a sphere of radius *r*. We denote by *S*′ the exterior *envelope* of all the spheres. For any point *p* ∈ *S*, there exists a point *q* ∈ *S*′ along the outward normal direction. Let *q*′ ∈ *S*′ be the intersection point of the ray $\vec{qp}$ and *S*′. Then the SDF at *p* is given by the penetration distance ‖*qq*′‖ − 2*r*. See Fig 2.

This seemingly minor change brings three significant benefits: First, it allows us to compute the inward-normal directions in a robust manner. Second, it greatly reduces the computation cost since at each point we set a single ray, rather than tens of rays. Moreover, by arranging the spheres into an oriented bounding box (OBB) tree [7], we can compute the penetration distances highly efficiently. Note that we don’t have the step of extracting the explicit representation of the offset surface. (Even if we have the offset surface, its hierarchical representation is still required for efficient computation of the intersection points between a ray and the offset surface.) Third, our method does not require watertight surfaces as the input—it supports both point clouds or general broken meshes possibly with noise or cracks.

Extensive computational results show that the offset-surface based SDF is robust to noise and insensitive to geometric details, and it also runs about 10 times faster than the existing method (note that we set a single ray, rather than tens of rays, at each point). We demonstrate the use of the new SDF in two typical applications including shape retrieval and shape segmentation.

## Related work

### Shape diameter function

Mathematically, SDF is closely related to the medial axis transform [1]. The major consideration of using shape *diameters*, rather than the medial surface, to shape analysis is for numerical reliability since the medial surface, in its nature, is sensitive to boundary changes. Combining the *extrinsic* SDF and the *intrinsic* geodesic centricity function (that measures the average geodesic distance from a vertex to all other vertices on the mesh), Gal et al. [2] proposed a pose-oblivious shape signature for shape retrieval. Taking advantage of the pose oblivious feature, Shapira et al. [3] applied SDF to construct consistent partitioning and skeletonisation across a family of objects. Such families may consist of either a single object in multiple poses and resolutions, or multiple objects which have a general common shape. Using SDF as the shape metric, Fan et al. [4] developed a painting-based tool for interactive mesh segmentation. Shapira et al. [6] applied SDF to partition an object into meaningful parts and finding analogous parts within other objects.

### 3D object retrieval

Shape signatures play a critical role in 3D object retrieval [8–12]. An effective signature should be able to capture the global geometric features and be invariant to affine transformation. It is also highly desired to be pose oblivious or even isometry invariant. Popular 3D shape signatures include Shape Context [13], Spin Image [14, 15], Shape Diameter Function [3], Heat Kernel Signature [12, 16], Wave Kernel Signature [17], and many others. SDF is broadly used in 3D object retrieval due to its simplicity and efficiency [5, 18]. When used for 3D object retrieval, a histogram which capturing the distribution of SDF is usually built for a 3D shape to extract a feature vector which can represent the 3D shape. Then the distance between two corresponding feature vectors are used to measure the difference between two 3D shapes.

### 3D mesh segmentation

Partitioning an object into meaningful parts is fundamental to many graphics applications. A typical segmentation algorithm constructs a shape signature by extracting some local and/or global geometric features, and then clusters the faces using the graph-cuts algorithm [19] where the Gaussian mixture model (GMM) is often used to define the probability for representing the presence of each triangle within a cluster. There is a large body of literature on 3D mesh segmentation. SDF can be not only directly used for 3D mesh segmentation [3], but also combined with other 3D shape signatures for 3D shape segmentation or co-segmentation. For example, SDF and other several shape signatures are fused and used for co-segmenting a set of 3D shapes by Wu et al. [20]. Besides, SDF is also used as one of the shape signatures and extreme learning machine is employed for segmenting 3D shapes in [21]. Wang et al. [22] and Meng et al. [23] use SDF and other shape signatures simultaneously to co-analysis or co-segment a set of 3D shapes. We refer readers to the state-of-the-art [21, 24] and the references therein.

### Surface offsetting

Mathematically, the offset surface can be viewed as the envelope of the set of spheres centered at the given surface and with a radius *r*. The offset surface for a single triangle has a slab-like shape. When the input surface is a triangle mesh, the offset surface can be obtained by a sequence of CSG operations over the slab-shaped basic shape elements. There are quite a few works on surface offsetting. Most of the existing methods [25–27] are based on volumetric representation or point representation [28, 29]. They build a signed distance field and then extract the iso-surface as the offset. Recently, Calderon and Boubekeur [30] defined morphological operators over an unorganized point sets based on point set surfaces and mathematical morphology. Zhou et al. [31] proposed a systematic solution to conducting a family of exact constructive solid geometry operations. For the problem discussed in this paper, however, there is no need to compute the offset surface. Even if the offset surface is known, we still have to build its hierarchical representation for efficient computation of the intersection points between a ray and the offset surface. Therefore, in this paper, we directly build the OBB representation of the offset surface (rather than extract its explicit representation first) and use it to the computation of SDFs.

### Bounding box tree

There are many choices of bounding structures. In this paper, we use oriented bounding boxes (OBB) [7] to facilitate the efficient computation of SDFs, rather than axis-aligned bounding boxes (AABB), to encode the rough volume occupied by the children nodes since OBBs are experimentally showed to have better performance for interference detection operations than AABBs. The two key components for the OBB tree construction include (1) finding a tight OBB for each geometric primitive and (2) grouping the OBBs, in a top-down style, into a tree hierarchy. We omit the details for brevity.

## An offset based approach for SDF

In this section, we first introduce a new definition of SDF based on the offset surface (with a given offset distance *r*). After that, we detail the OBB based representation of the offset surface that facilitates the efficient computation of SDF. Note that different from the traditional OBB tree, each leaf node encodes a sphere, or a cylinder, or a tri-prism that can be represented by a simple algebraic equation array.

### New definition of SDF based on the offset surface

Given a surface *S*, typically a polygonal mesh in the discrete setting, the offset surface *S*′ in a distance *r* can be defined to be the envelope of the sphere set

$$\begin{array}{c} \{O(p,r)|p\in S\}, \end{array}$$

where O(p,r) is the sphere centered at *p* and has a radius *r* (generally less than a given tolerance). It is easy to know that if *S* is an orientable and closed 2-manifold, the offset surface *S*′ generally has two layers, one lying inside *S* and one outside *S*. The layers enclose a connected body with a thickness 2*r*. The exterior layer of *S*′ is of our interest.

Next we shall introduce the definition of SDF based on the offset surface *S*′. As Fig 2 shows, for any point *p* ∈ *S*, there exists a point *q* on the offset surface such that *q* is the first intersection point along the outward normal direction. We then intersect the offset surface with the ray $\vec{qp}$ and find the other intersection point *q*′. We take the distance between *q* and *q*′ as the penetration distance into *S*′, denoted by *D*. The SDF at *p* is then defined to be *D* − 2*r*.

However, we don’t need to actually compute the offset surface since the key to support fast query of intersections between the offset surface *S*′ and a ray lies in building a hierarchical representation of *S*′. Therefore, we next discuss how to build the OBB tree of *S*′ without explicitly extracting *S*′.

**Fig 3:**
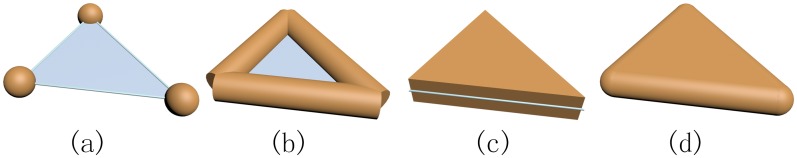
The volume. (d) enclosed by the offset surface of a triangle is the union of three spheres (a), three cylindrical shapes (b) and one tri-prism (c).

Fig 3 shows the simplest situation where the polygonal mesh *S* consists of a single traingle △*ABC*. It’s easy to know that the volume enclosed by the offset surface of △*ABC* at a distance *r* is the union of the following three types of basic geometric elements, as achieved in [32]:

- 3 spheres O(A,d),O(B,d),O(C,d).
- 3 cylindrical shapes with *AB*, *BC*, *CA* as center lines.
- 1 tri-prism centered at △*ABC*.

The sphere centered at *A* (resp. *B* or *C*) is given by the equation ‖*x* − *A*‖ ≤ *r* (resp. ‖*x* − *B*‖ ≤ *r* or ‖*x* − *C*‖ ≤ *r*), the cylindrical shape along $\vec{AB}$ is given by the equation $\|\left(x-A\right)\times \vec{AB}\left\|\leq r\right|\vec{AB}|,A\cdot \vec{AB}\leq x\cdot \vec{AB}\leq B\cdot \vec{AB}$, and the tri-prism can be represented by

$$\begin{array}{cc} \left|\text{Det}\left(x-A,B-A,C-A\right)|-r\right|\vec{AB}\times \vec{BC}| & \leq 0,\\ {}[(x-A)\times \vec{AB}]\cdot \left[\left(C-A\right)\times \vec{AB}\right] & \geq 0,\\ {}[(x-B)\times \vec{BC}]\cdot \left[\left(A-B\right)\times \vec{BC}\right] & \geq 0,\\ {}[(x-C)\times \vec{CA}]\cdot \left[\left(B-C\right)\times \vec{CA}\right] & \geq 0. \end{array}$$

Furthermore, it’s pretty fast to get the oriented bounding box for basic geometric element, or compute the intersection points between a basic geometric element and a straight line.

For a general polygonal mesh, we can build an OBB tree with each leaf node being a basic geometric element (sphere, cylinder or tri-prism). Suppose that the tree contains *N* basic geometric elements *G*_1_, *G*_2_, ⋯, *G*_*N*_. The following three types of operations are required to support the computation of SDF. Obviously, they can be greatly speeded up by the OBB tree of basic geometric elements. (Note that we don’t need to compute the explicit form of the offset surface.)

- *Checking if a point is located on the offset surface.* A point *p* is located on the offset surface if and only if (1) *p* cannot be located completely inside any basic geometric element, and (2) there exists a basic geometric element *G*_*i*_ such that *p* is located on *G*_*i*_’s surface.
- *Projecting a point onto the offset surface.* If *p* is located in the exterior space of the offset surface, then we simply project *p* to the geometric element that is nearest to *p*. Otherwise, we omit those geometric elements that don’t contain *p* and project *p* to *G*_*i*_ with the minimum distance to move *p* out of the basic element.
- *Intersecting the offset surface with a ray.* It can be achieved by intersecting the ray with the geometric elements arranged in the OBB tree and then identifying those points located on the offset surface.

In case where *S* has small gaps or small holes or even is a point cloud, the offset surface *S*′ may be no longer of two layers and in this case the volume enclosed by *S*′ cannot separate the 3D space into interior and exterior regions. That is to say, the above definition doesn’t work since a ray may pass through a gap of *S*′. However, we observe that if the gaps are sufficiently small or the sample points are sufficiently dense, then the surface *S*′ consists of two layers that enable the SDF to be established. We state this observation using the following theorem.

**Theorem 0.1** *Let P be an ϵ*-*dense point cloud that represents an orientable and closed surface S, i.e. for any point s* ∈ *S, there always exists a point p* ∈ *P such that* ‖*s* − *p*‖ ≤ *ϵ*. *Then the volume enclosed by the offset surface in a distance r is able to separate* $R^{3}$ *into two disconnected regions if r* > *ϵ*.

**Proof.** Given a point *s* ∈ *S*, there always exists a point *p* ∈ *P* such that ‖*s* − *p*‖ ≤ *ϵ*. The point *s* lies inside the sphere O(p,r) since *r* > *ϵ*. Due to the arbitrariness of *s*, the surface *S* lies completely inside the union of a set of spheres {O(p,r)∣p∈P}. Since *S* is orientable and closed, we conclude that the volume enclosed by *S*′ is able to separate $R^{3}$ into disconnected regions.

**Remark:** Note that in practice it is possible that the interior region separated by *S*′ is empty (when the volume bounded by *S* is very thin while the offset distance *r* is quite large). In this case, the SDF is still well defined since we only care about the outer layer of the offset surface.

### Algorithm

Once the OBB tree has been constructed, it’s pretty fast to report the penetration distance of a ray. Two kinds of operations are central to compute SDFs. One is to find closest points to the offset surface and the other is to compute intersection points between a ray and a box, or a sphere or a cylinder or a tri-prism. Let *p* be a point on the input surface *S*, as shown in Fig 2. First, we need to find the closest point *q* on the offset surface. The point *q* has to meet the following condition:

$$\begin{array}{c} \frac{\vec{qp}\cdot \text{Normal}\left(q\right)}{\|\vec{qp}\|}>1-\epsilon , \end{array}$$ (1)

where Normal(*q*) is the unit normal to the corresponding geometric element that contains *q*, which is to guarantee that *q* is located on the outer offset surface. And the penetration distance by the ray $\vec{qp}$ into *S*′ can be computed by finding the intersection point *q*′ that is closest to *q*. Similarly, let *p*′ ∈ *S* be the closest point to *q*′ ∈ *S*′ and we specify the following requirement on *q*′:

$$\begin{array}{c} \frac{\vec{q^{\prime }p^{\prime }}\cdot \text{Normal}\left(q^{\prime }\right)}{\|\vec{q^{\prime }p^{\prime }}\|}>1-\epsilon . \end{array}$$ (2)

The SDF at *p* is then given by ‖*qq*′‖ − 2*r*. The pseudo-code to support the SDF query is shown in Algorithm 1.

**Algorithm 1** Query the SDF at *p* ∈ *S*.

1: **procedure** QuerySDF(*p*, *r*)

2:  Find the closest point *q* ∈ *S*′ to *p* with the support of the OBB tree;

3:  Find the point *q*′ ∈ *S*′ such that *qq*′ defines the penetration segment into *S*′;

4:  **return** ‖*qq*′‖ − 2*r*.

5: **end procedure**

## Evaluation

We implemented and experimented with our algorithm on a computer with a 64-bit version of Win7 system, a 3.07 GHz Intel(R) Core(TM) i7 CPU and 6 Gb memory. The coding language is C++. For the original SDF, the opening angle is set to *π*/3 and the number of rays inside each normal cone is 25. For our algorithm, the offset distance, without specification, is taken to be 5% of the bounding box size. In the following, we shall use extensive experimental results to exhibit the high performance and robustness of our algorithm. The resulting SDF is experimentally shown to be pose oblivious and invariant to noise, cracks and geometric details.

### Performance

**Fig 4:**
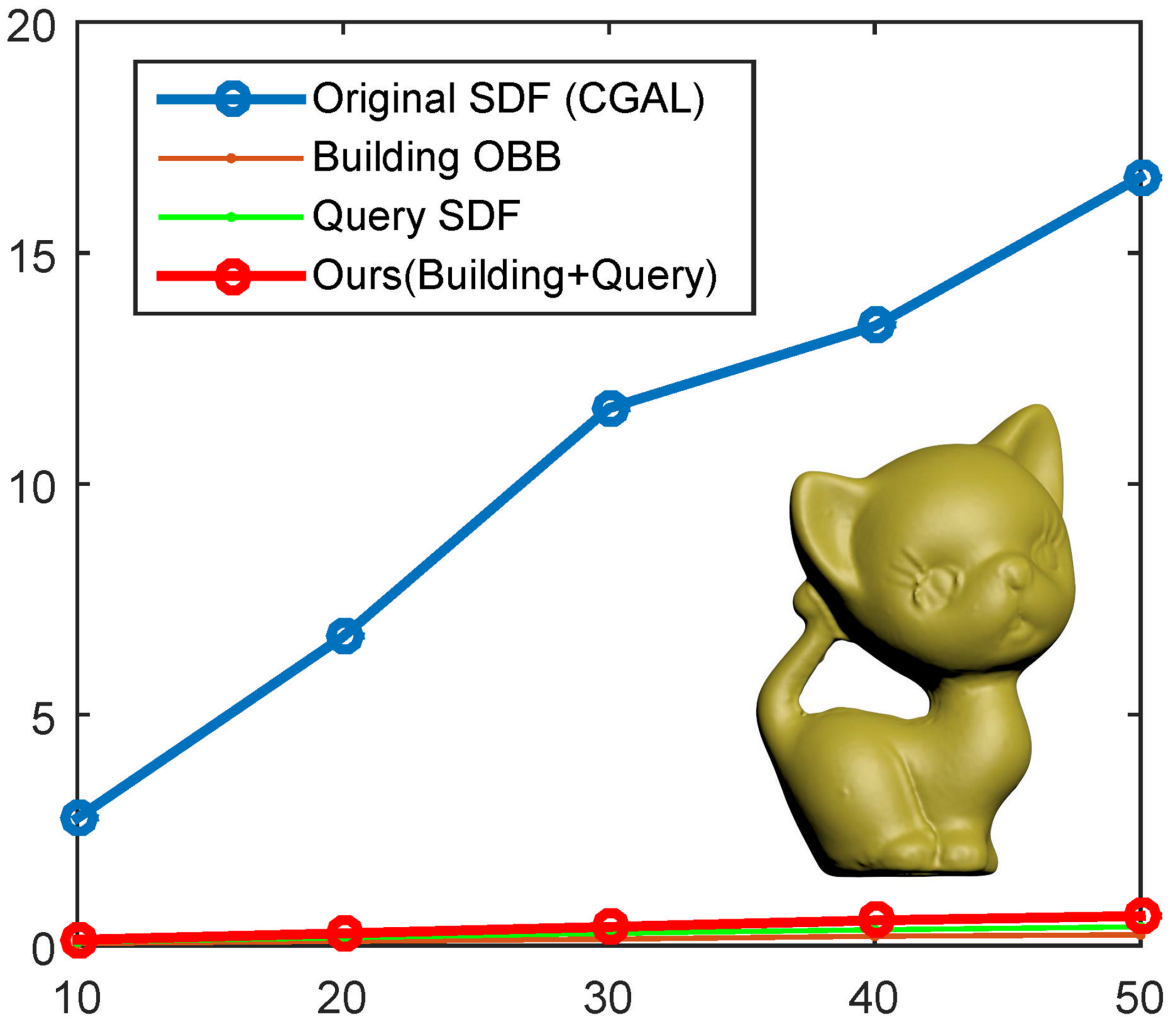
We test the performance on various resolutions of the Kitten model. The blue plot shows that the time cost of the original SDF, while the red plot shows that of our SDF. Here the total timing includes two parts, one for building the OBB tree and the other for querying the SDF over the input surface. Note that the implementation of the original SDF is based on CGAL 4.7.

As shown in Fig 4, we plot the timing data on various resolutions of the Kitten model that has 10K to 50K faces. The timing of our algorithm basically consists of two parts, one part for building OBB and the other for querying the SDF on the mesh surface. CGAL 4.7 is able to compute the raw SDF values and thus we get the timing statistics of the original SDF based on the CGAL implementation. From the performance plots, it can be seen that our algorithm runs faster, by one order of magnitude, than the conventional SDF algorithm. An obvious difference is that the conventional SDF algorithm requires a number of rays inside the inward normal cone, typically 25 rays, to find the penetration distance when evaluating the SDF at a point while our SDF algorithm needs only a single ray to get the SDF, which accounts for the significant performance improvement. A timing statistics table of quantitative comparison is available in Table 1, where the test models are from the SHREC′11 3D shape dataset.

**Table 1:** Timing statistics of the original SDF algorithm and our SDF algorithm, where the time cost is measured in seconds.

Model Name	#Faces	Original SDF	Our SDF
Alien	18K	5.796	0.611
Ant	19K	7.270	0.767
Armadillo	19K	6.812	0.643
Bird1	19K	7.834	0.824
Bird2	8K	2.659	0.279
Camel	18K	5.846	0.544
Cat	19K	6.033	0.581
Centaur	17K	7.342	0.738
Dino_skel	19K	7.745	0.820
Dinosaur	19K	6.893	0.735
Dog1	19K	6.994	0.665
Dog2	18K	6.117	0.658
Flamingo	19K	6.070	0.652
Glasses	19K	5.696	0.568
Gorilla	18K	6.019	0.631
Hand	19K	6.466	0.610
Horse	19K	6.681	0.660
Lamp	19K	7.026	0.744
Man	19K	6.362	0.665
Octopus	19K	7.992	0.845
Paper	19K	8.407	0.856
Pliers	19K	5.577	0.511
Rabbit	19K	8.204	0.855
Santa	19K	6.907	0.734
Scissor	18K	4.946	0.512
Shark	15K	4.228	0.448
Snake	17K	4.060	0.430
Spider	19K	6.216	0.610
Twoballs	19K	8.087	0.824
Woman	18K	6.729	0.640

### Robust normal estimation

**Fig 5:**
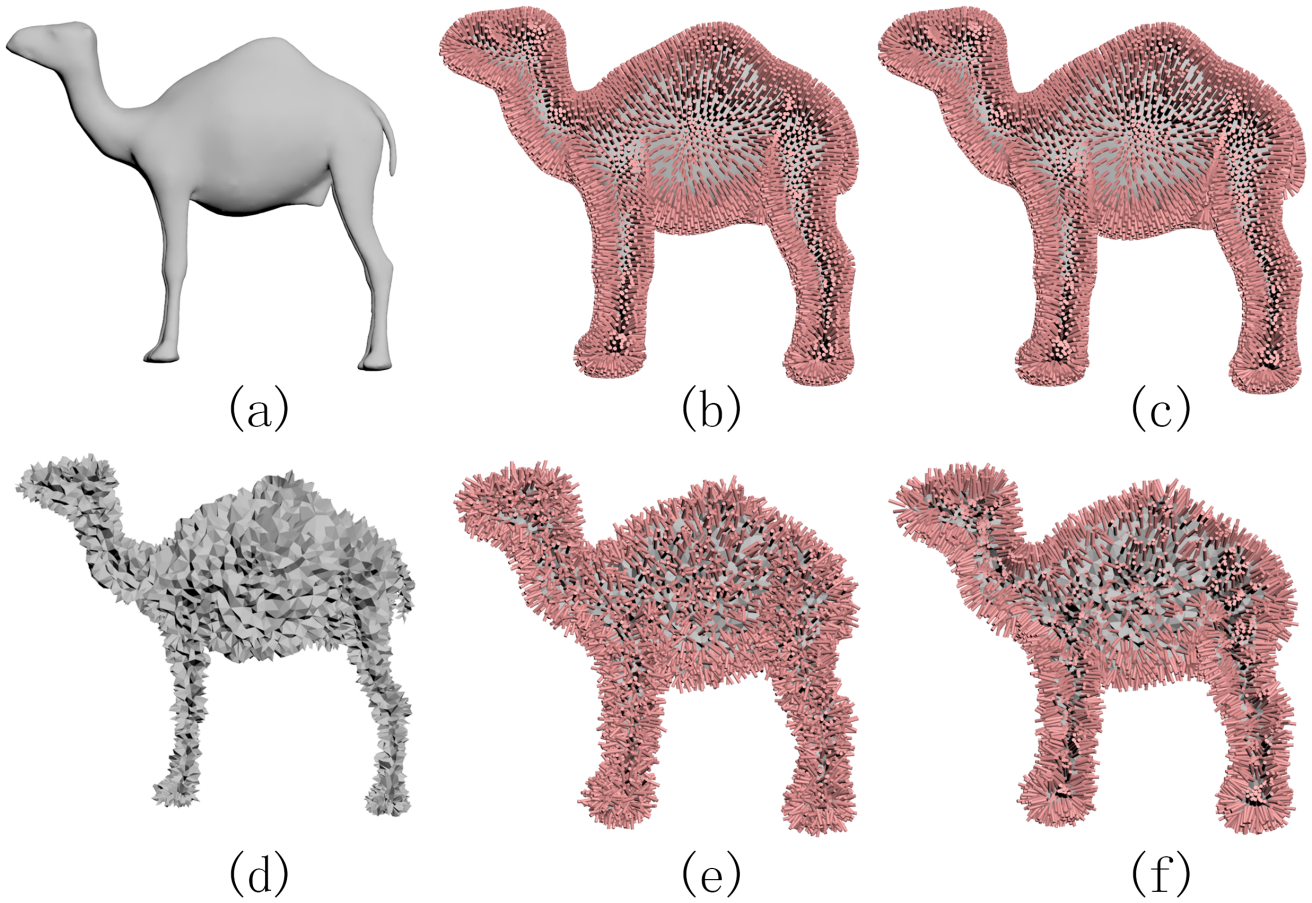
Traditionally we can estimate the normal vector at a vertex *v* by averaging the normals to the incident faces. When the input is a smooth model (a), both the traditional method (b) and our offset based method (c) can give a desirable estimation. However, if the input surface has serious noise (d), the offset based technique is still able to give a robust estimation of normals (f), while the traditional method cannot (e).

Numerous geometry processing tasks, such as mesh reconstruction and mean curvature estimation, require the knowledge of the surface normals. Unlike continuous surfaces, point clouds and polygonal meshes lack an explicit form of normals. Most of the excising approaches depend on the geometric edges or vertices and thus are prone to artifacts. Based on the offset technique, we can estimate normals like this: Let *p* be a point on the input surface and *q*, lying on the offset surface, be the closest point to *p*. We estimate the normal at *p* according to the directional segment $\vec{pq}$. Taking Fig 5 as an example, we can see that when the input is a smooth model, both the traditional method (estimating the normal vector at a vertex *v* by averaging the normals to the incident faces to *v*) and our offset based method can give a desirable estimation. However, if the input surface has serious noise (perturbing each vertex of the original model randomly with a 6% position movement with regard to the size of the bounding box), it is highly non-trivial to define the normals to surface in a robust style. In spite of this, Fig 5 shows that the offset based technique is still able to give a robust estimation of normals, while the traditional method cannot.

### Insensitivity to noise/cracks

**Fig 6:**
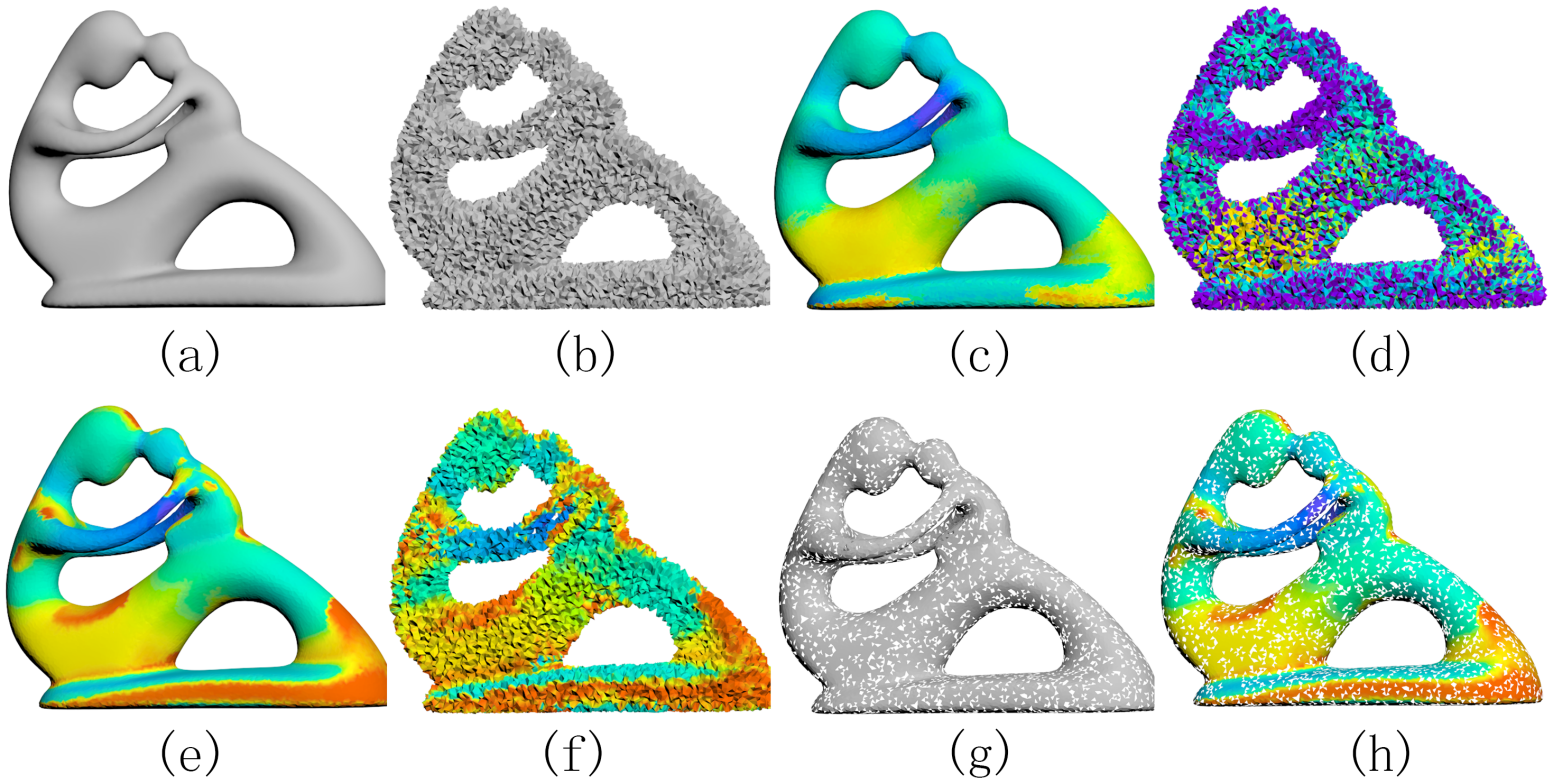
Insensitivity to noise/cracks. The smooth Fertility model is shown in (a), while the noisy version is in (b). The original SDF algorithm (c-d) is sensitive to noise and cannot be applied to shapes with cracks (g), while our SDF algorithm obtains a similar SDF (e) even if the input model has noise (f) or cracks (h).

It’s quite often that the input model has serious noise/cracks especially in engineering. Therefore, the property of insensitivity to noise/cracks is central to deal with these models. In Fig 6, we use three Fertility models to test our SDF algorithm, as well as the original SDF algorithm. In order to get a version with noise, we perturb each vertex of the smooth model with random noise (perturbing each vertex of every triangle randomly with a 6% position movement with regard to the size of the bounding box). The model with cracks is obtained by randomly removing 15% triangles of the surface. From the visual contrast, it can be seen that even when we introduce large noise into the input model, our algorithm can still get a nearly invariant SDF. Intuitively, since we offset the input surface outward in a distance, the resulting offset surface is generally smoother and has less geometric details than the input one, which finally leads to a SDF that is insensitive to noise. In addition, when the input models has some cracks, our algorithm works well since Theorem 0.1 asserts that the underlying offset surface is still watertight as long as the area of the cracks is limited.

### Invariance to geometric details

**Fig 7:**
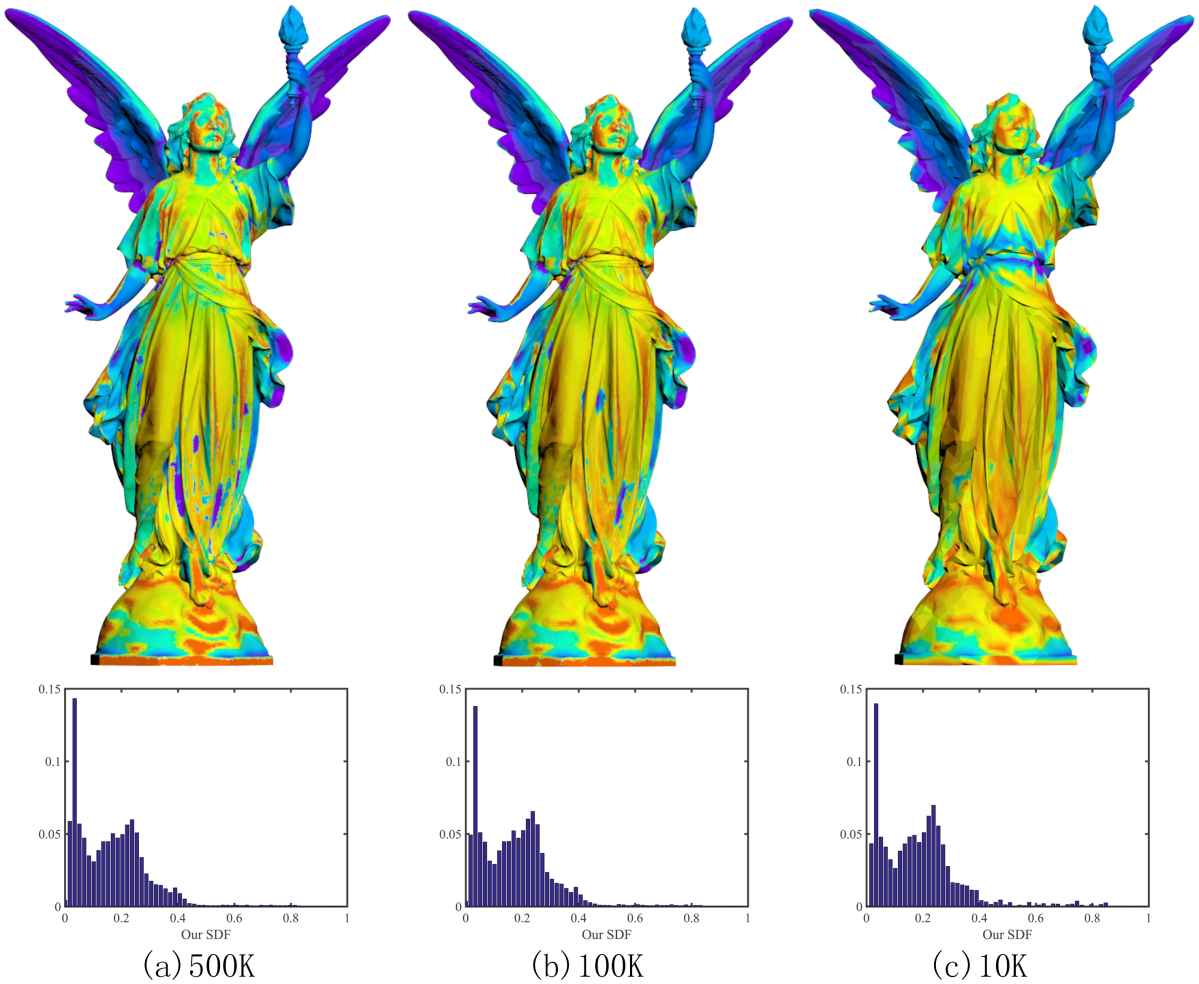
Invariance to geometric details. Although we simplify the original Lucy model from 500K triangles (a) to 100K triangles (b) and 10K triangles (c) respectively, the SDFs, as well as their corresponding histograms, don’t have a significant difference.

Mesh simplification is a common operation in computer graphics. Generally speaking, geometric features are kept but geometric details are missing during mesh simplification. As Fig 7 shows, the three versions of the Lucy model respectively have 500K triangles, 100K triangles and 10K triangles. They obviously have different amount of geometric details. In spite of this, their SDFs don’t have a significant difference. Here Further experimental results show that our SDFs vary continuously and monotonically with regard to local shape changes. These nice geometric properties imply that the new SDF induces a more stable shape signature, which is very useful to shape analysis; See its applications in the next section.

### Pose oblivious

**Fig 8:**
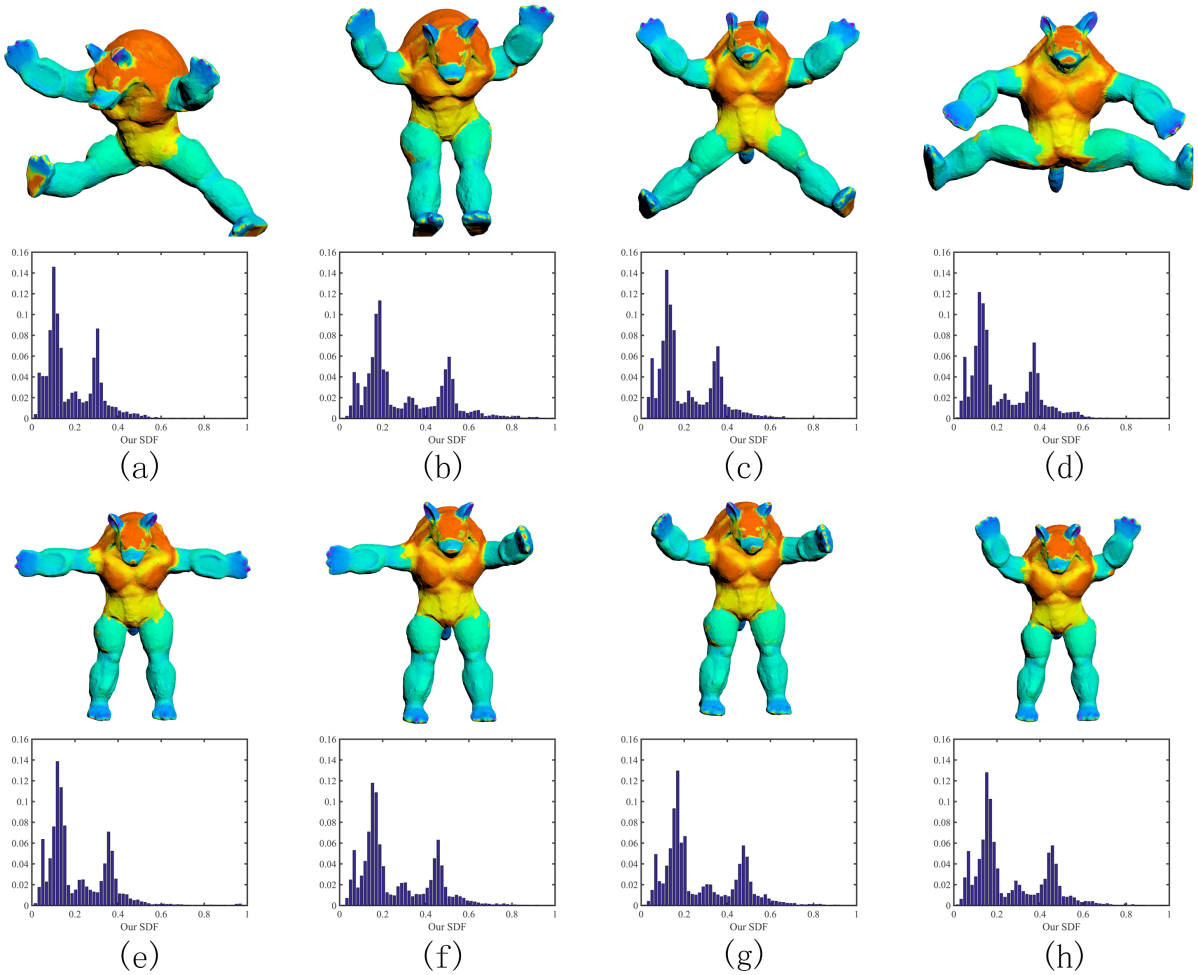
Independence of pose changes. We obtain consistent SDFs on various poses of the Armadillo model. Thus, the histogram of the SDF can be used as a shape signature.

We shall show the proposed SDF in this paper is independent of pose changes and therefore able to define an effective signature and get consistent partitioning of 3D shapes. As Fig 8 shows, in spite of various poses, the Armadillo models have quite similar SDFs by our algorithm. In fact, the geometric intuition behind the SDF is that it is related to the medial axis transform in its nature and serves as a link between the object’s volume and its boundary, mapping volumetric information onto the surface boundary mesh, as Shapira et al. [3] observed. Our offset based technique provides an alternative way to compute the penetration distance and therefore still inherits this spirit.

### Applicable to point clouds

**Fig 9:**
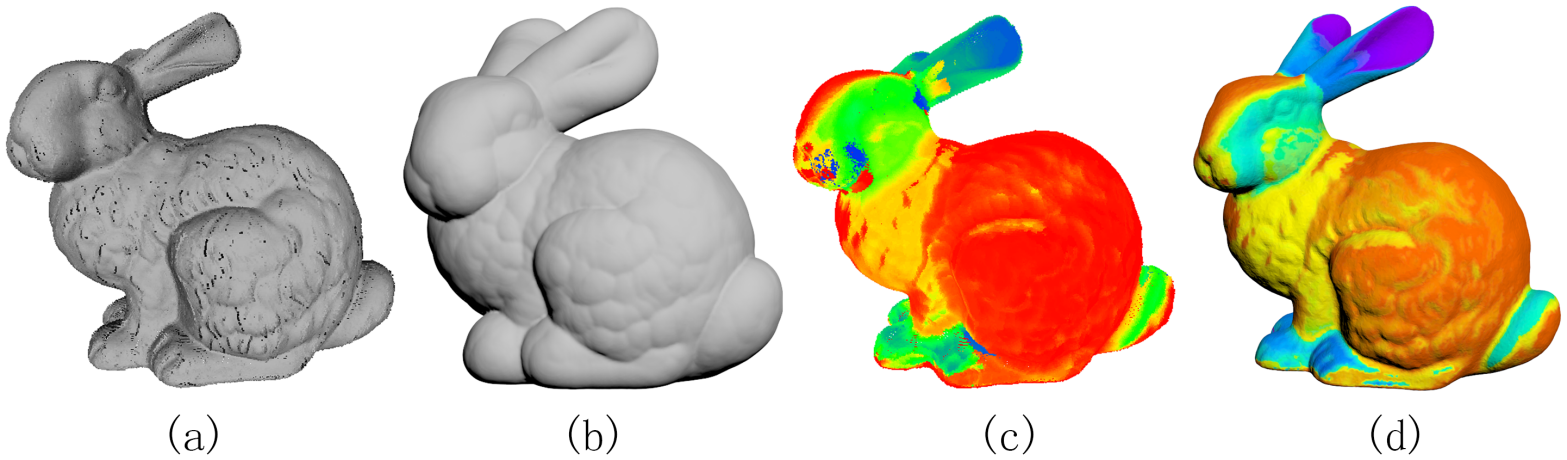
SDF on point clouds. For the point cloud of the Stanford Bunny (a), we first build the OBB tree to accommodate the collection of spheres (b), each centered at a point of the point cloud, and then compute the SDF of the point cloud (c) based on the outer envelope of the sphere set. It’s quite close to the SDF (d) on the complete mesh. Note that the (a) and (c) are rendered in Meshlab while (b) and (d) are rendered in 3ds Max, and therefore they have different visual styles.

Digital 3D models captured from modern devices often have cracks and gaps. Generally they have to experience a tedious lifecycle to become a perfect 3D model, typically a polygonal mesh with good triangulation. A common kind of imperfect models is point clouds. Therefore, it is pretty useful if we can extend the SDF algorithm to point clouds. Recall that that the key component of our algorithm is to compute the OBB tree of the sphere set centered at each point belonging to the input point cloud. As Theorem 0.1 points out, as long as the offset distance is sufficiently large, we can guarantee that the outer layer of the envelope is closed, in which case the SDF is well defined. Taking Fig 9 for an example, we come to compute the SDF on the original point cloud of the famous Stanford Bunny. It’s noted that there are two holes at the bottom. By taking the offset distance to be 5% of the bounding box size, the union of the sphere set centered at the point cloud is able to separate $R^{3}$ into two parts, which ensures the SDF to be computed. Furthermore, the SDF on the point cloud is quite close to the SDF on the complete Bunny mesh. More generally, our algorithm can deal with triangle soups or defect models with gaps and cracks.

## Applications

Since the SDF is invariant to pose changes, it has been found useful in many applications, as mentioned in [3] and [6]. In this section, we use two application occasions, i.e. shape retrieval and segmentation, to demonstrate the advantage of the SDF proposed in this paper over the original SDF.

### Experimental setting

Our tests of shape retrieval and segmentation were made on the SHREC’11 non-rigid 3D shape dataset [33], which is open to the computer graphics community for public tests. The dataset contains 600 shapes that can be categorized to 30 classes, each containing 20 shapes. To ensure the invariance to scaling, the total surface area of each input model is normalized to 1 as a preprocessing.

### Shape retrieval

Generally, the first step of shape retrieval is to collect one or more signatures that encode the shape of interest into a scalar or vector field over the surface. The dissimilarity between two 3D shapes is then defined to be the distance between their signatures. For a database of *N* shapes, the *N* × *N* all-pair distance matrix is used to measure the retrieval performance of the given signature.

More precisely, if the signature is a scalar field over the mesh surface, it needs to be converted a histogram, represented by a number of bins, to facilitate distance computation. For a vector-field signature, however, it’s common practice to compute the histogram depending on the bag-of-feature (BOF) technique. In this paper, both the number of bins and that of the feature bags are both experimentally set to be *B* = 80, and we use 1-norm to define the distance.

A weighting scheme is also needed when generating histograms. Generally, if the signature is defined on vertices, then the scalar or vector defined at vertex *v* is weighted by the Voronoi area dominated by *v*. Correspondingly, if the signature is defined on the face set, then the scalar or vector defined at face *f* is weighted by the area of *f*.

Next, we shall compare the retrieval performances of the original SDF algorithm and our SDF algorithm on the well-known SHREC’11 3D shape dataset. The signatures used here include:

- The conventional SDF [2];
- Our proposed SDF (the offset distances are respectively set to 1%, 3% and 5% of the bounding box size);
- Concatenating the above three scalar fields into a vector-field signature.

In the field of information retrieval, *Precision-Recall* curve is a commonly used tool to measure the retrieval precision. Suppose that there exist *K* objects in a certain class G. If *m* objects that don’t belong to G are contained in the retrieval list in order to extract the objects in G with the percentage of *x*, then its retrieval precision is defined as

$$\begin{array}{c} P\left(x\right)=\frac{Kx}{Kx+m},\;x\in \left[0,1\right]. \end{array}$$

**Fig 10:**
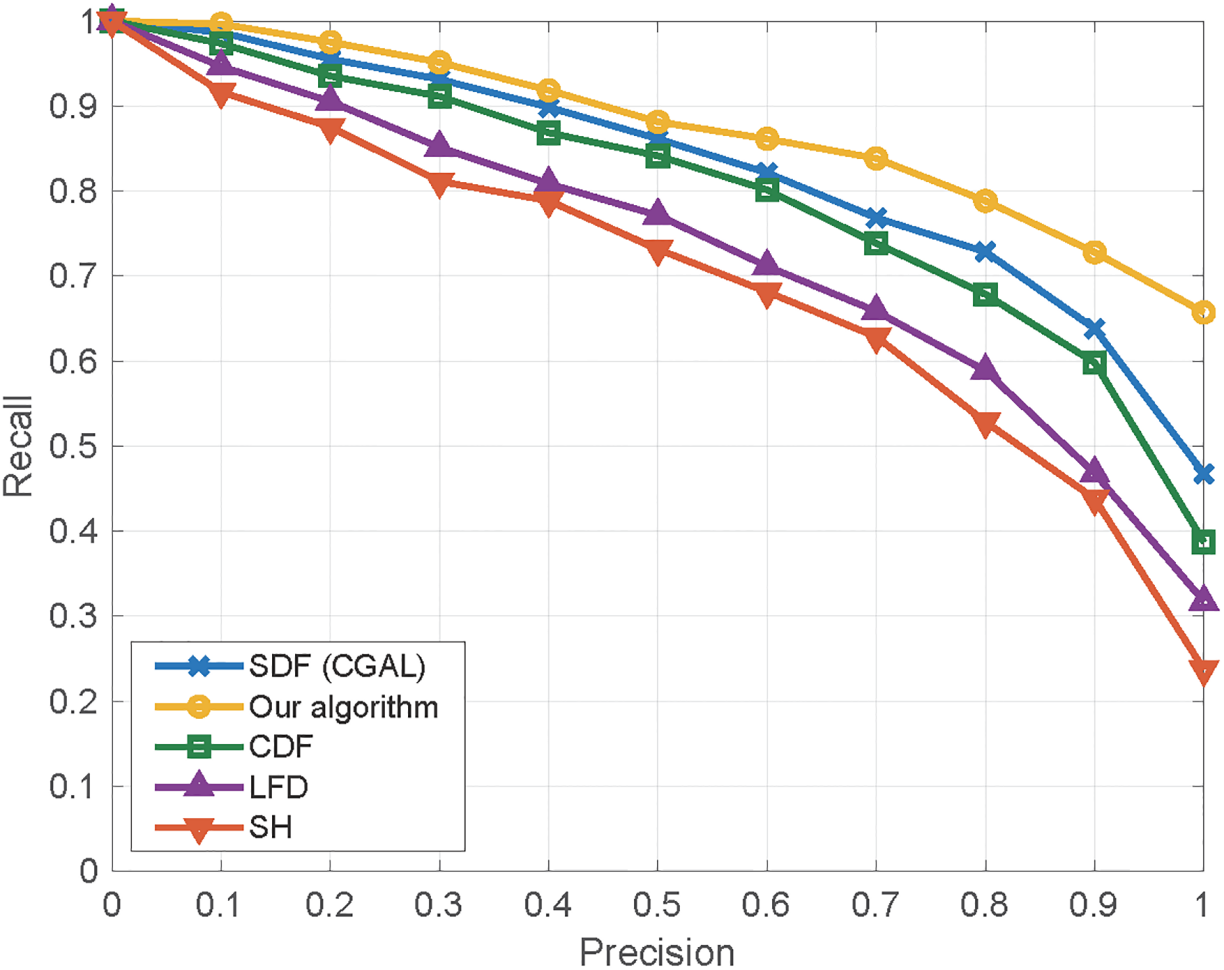
Precision-Recall plots on SHREC’11 3D shape dataset.

*P*(*x*) is generally a monotonically decreasing curve. The superior curve means higher retrieval performance. Fig 10 shows the performance plots of the above signatures. We can see that whatever the offset distance (1% to 5%) is, the SDF algorithm proposed in this paper has a better retrieval performance than the conventional SDF algorithm. Furthermore, concatenating our SDF values together generates a better signature that outperforms the conventional SDF algorithm and the offset based SDF algorithm using an individual offset distance.

We also use the following five measures to quantitatively compare the conventional SDF algorithm and our algorithm on the SHREC’11 3D shape dataset. These measures include [8, 9, 34]:

- **Nearest neighbor (NN):** the percentage of the closest matches belonging to the same class as the query.
- **First-tier and second-tier:** the percentage of models in the query’s class that appear within the top *K* − 1 and 2(*K* − 1) matches respectively, where *K* is the size of the query’s class.
- **E-measure:** a composite measure of the precision and recall for a fixed number (32) of retrieved results.
- **Discounted Cumulative Gain (DCG):** a statistic that weighs correct results near the front of the ranked list more than correct results toward the end of the list [35].

The statistics of various measures are shown in Table 2, from which we can see that our algorithm has an advantage of shape retrieval over the previous SDF algorithm. Fig 10 shows the PR plot of the related algorithms including the original SDF, our SDF, CDF [2], Light Field Descriptor (LFD) [36] and Spherical Harmonics (SH) [37].

**Table 2:** Retrieval performance comparisons on SHREC’11 3D shape dataset using various measures.

	NN	Tier 1	Tier 2	E-Measure	DCG
Our SDF (*d* = 1%)	94.50%	50.95%	59.45%	43.69%	82.76%
Our SDF (*d* = 3%)	93.67%	51.76%	60.77%	44.83%	82.98%
Our SDF (*d* = 5%)	92.83%	51.27%	60.58%	44.44%	82.53%
Our SDF (Concatenation)	96.33%	53.56%	61.80%	45.44%	84.05%
Conventional SDF	94.33%	48.08%	57.98%	42.14%	81.56%

### Shape segmentation

Shape segmentation has been widely studied in the last several years. It’s an increasingly common practice to take a signature as the input and then use the clustering and graph cuts techniques to produce the final segmentation results. In the first step, we over-segment each shape in the shape dataset into *L* (50 in this paper) primitive patches, like that achieved in the super-pixel based image segmentation [38, 39]. The choice of *L* depends on experience, generally a balance between performance and segmentation accuracy. Next, we build a feature vector for each patch by capturing the signature distribution on this patch. After that, we use the Gaussian mixture model to compute a probability matrix to encode the presence of each patch within a cluster. Finally, by using the graph cuts algorithm [19], we can segment the input model into a number of surface patches.

In the computer graphics community, the Rand Index defined by [40] is very popular in measuring the similarity between two segmentation results of the same shape and the common way to evaluate the overall performance of a shape segmentation algorithm is to compare the segmentation results against the handcraft ground-truth. However, the shape segmentation ground-truth of SHREC’11 is not available yet, to our best knowledge. Therefore during the last three months, we asked 7 graduate students to independently segment all the 600 shapes in SHREC’11, like that achieved in [40].

**Fig 11:**
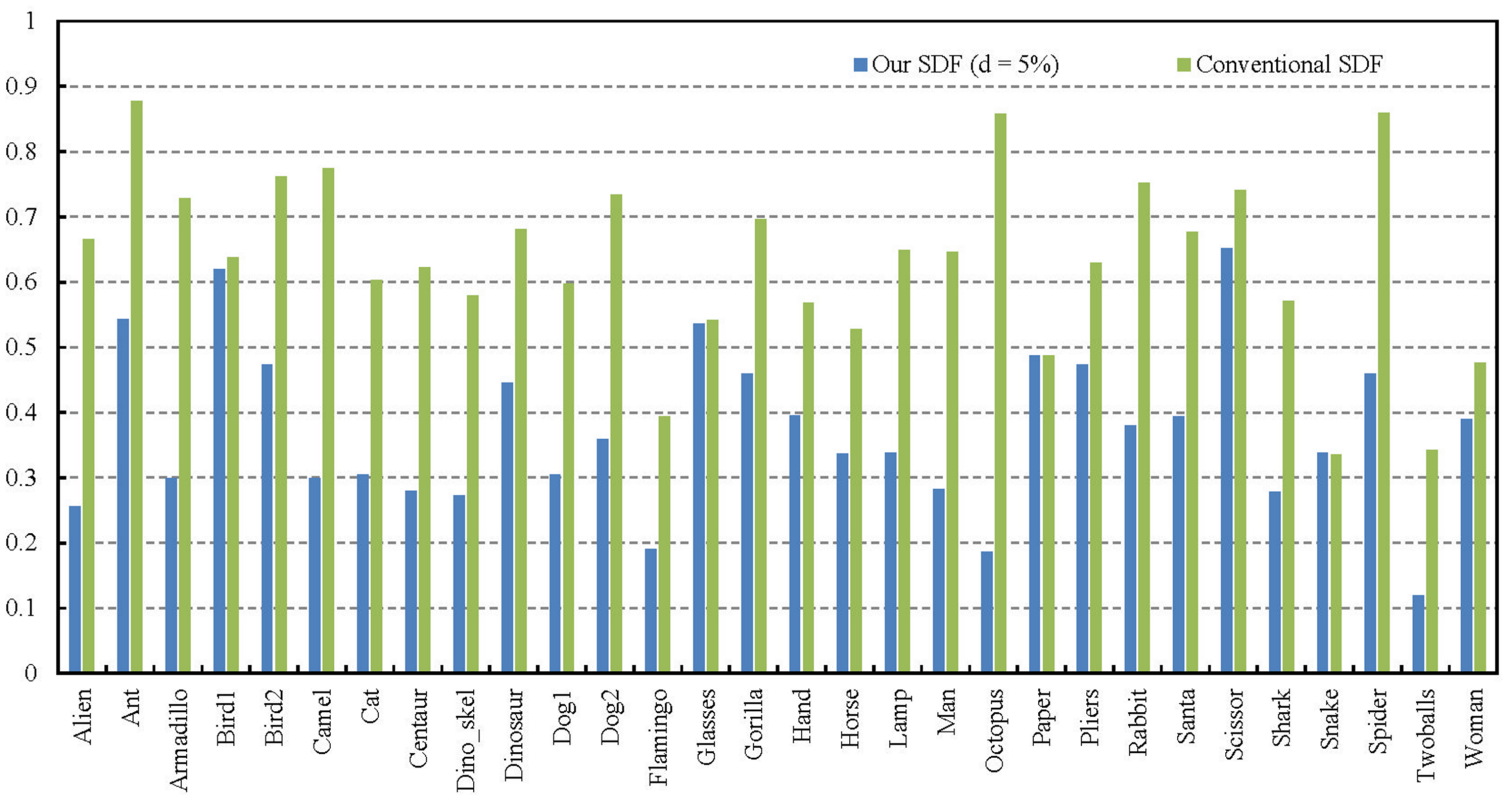
Quantitative comparisons among the segmentation results. We use average Rand Index scores to compare the overall segmentation performances of our 5%-offset SDF and the conventional SDF on SHREC’11. Note that lower values indicate closer similarity to the human-generated ground truth.

**Fig 12:**
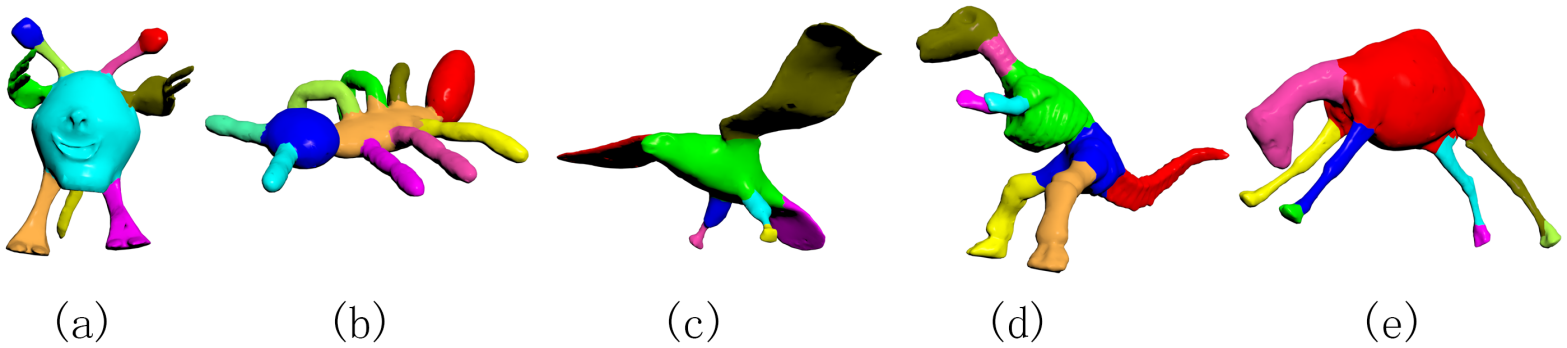
Representative segmentation results produced by our algorithm.

Fig 11 shows the quantitative comparisons among the segmentation results of our 5%-offset SDF and the conventional SDF on SHREC’11. The Rand Index scores show that our algorithm has a higher segmentation performance over the conventional SDF algorithm. We also show the average Rand Index scores over all categories in Table 3. The statistics exhibit that our SDF algorithm significantly outperforms the conventional SDF algorithm. Some representative segmentation examples by our algorithm is shown in Fig 12.

**Table 3:** Segmentation performance comparisons using Rand Index scores on the SHREC’11 3D shape dataset.

	Our SDF (*d* = 5%)	Conventional SDF
Average Rand Index score	0.3719	0.6339

## Conclusion

In this paper, we propose an offset based technique, yet without really computing the offset surface, to define and compute SDF. Our SDF is insensitive to noise (or even geometric details) and able to deal with point clouds, runs one order of magnitude faster than the conventional SDF, and exhibits a superior performance over the conventional SDF when applied to shape retrieval and shape segmentation. Two main features of our algorithm that distinguish itself from the original SDF include (1) the offset surface is able to give a better estimate of normals to the input surface and (2) only a single ray is required to estimate the SDF at a point, which accounts for the significant performance improvement.

The proposed SDF has several limitations, which are worth of further investigation. First, we observe there are some cases where the resulting SDFs are negative (slightly less than zero). Second, the choice of the favorite offset distance depends on our experience and is not self-adaptive yet. Finally, the SDF, if enforced several bilateral filtering iterations, may lead to better retrieval performance. We will further study these problems in the near future.
